# CD8^+^ T Cell Responses to *Plasmodium* and Intracellular Parasites

**DOI:** 10.2174/1573395509666131126232327

**Published:** 2013-08

**Authors:** Nicolas Villarino, Nathan W. Schmidt

**Affiliations:** Department of Microbiology, University of Tennessee, Knoxville, TN 37996, USA

**Keywords:** CD8^+^ T cell, immunity, *Leishmania*, malaria, *Plasmodium*, protozoa, *Toxoplasma*, *Trypanosoma*.

## Abstract

Parasitic protozoa are major threats to human health affecting millions of people around the world. Control of these infections by the host immune system relies on a myriad of immunological mechanisms that includes both humoral and cellular immunity. CD8^+^ T cells contribute to the control of these parasitic infections in both animals and humans. Here, we will focus on the CD8^+^ T cell response against a subset of these protozoa: *Plasmodium, Toxoplasma gondii, Leishmania and Trypanosoma cruzi*, with an emphasis on experimental rodent systems. It is evident a complex interaction occurs between CD8^+^ T cells and the invading protozoa. A detailed understanding of how CD8^+^ T cells mediate protection should provide the basis for the development of effective vaccines that prevent and control infections by these parasites.

## INTRODUCTION

The parasitic protozoa, *Plasmodium *spps*., Toxoplasma gondii, Leishmania *spps*. *and *Trypanosoma cruzi *are medically important pathogens around the world, causing significant disease in humans. Resistance and control of infections by these parasites relies on a competent immune system with multiple immunological mechanisms contributing. *Plasmodium spps., Toxoplasma gondii, Leishmania spps. *and *Trypanosoma cruzi *are intracellular parasites in both humans and mice. As a consequence of the intracellular infection these parasites are susceptible to immune mediated control by CD8^+^ T cells, which target intracellular pathogens. Indeed, there is strong evidence that CD8^+ ^T cell responses are an important component of the host defense mechanism against these parasites. However, the degree of protection afforded by CD8^+^ T cells against these pathogens depends on the parasite, and may also differ among species of a given protozoa. Despite the induction of a robust immune response, these protozoa can delay or prevent immune clearance, thus, allowing the establishment of chronic infection in the host. Here we will review the contribution of CD8^+^ T cells during *Plasmodium*, *T. gondii*, *Leishmania *and *T. cruzi *infections. A more thorough understanding of CD8^+^ T cell responses against these intracellular parasites may be applicable to other intracellular parasites. Furthermore, there are no licensed vaccines that target these parasites through the induction of protective CD8^+^ T cells. Therefore, there is a need for continued research on understanding CD8^+^ T cell responses against these parasitic infections.

## GENERATION OF *PLASMODIUM*-SPECIFIC CD8^+^ T CELL RESPONSES

*Plasmodium* infections in humans and rodents begin when an infected female *Anopheles *mosquito injects saliva containing *Plasmodium* sporozoites into the dermis during a blood meal. After deposition of sporozoites into the skin, the parasites enter host blood vessels where they travel to the liver and establish infection in hepatocytes. In the case of both humans and rodents, the initial liver stage of infection is relatively short lived [1, 2], resulting in little if any opportunity for the host to mount CD8^+^ T cell responses that are capable of eliminating infected hepatocytes during the initial infection. However, using the rodent model of malaria it has been shown that memory CD8^+^ T cells recognize parasite-infected hepatocytes upon re-exposure to the parasite, and are capable of preventing the parasite from progressing into the erythrocytic stage of infection [3, 4].

Following inoculation of sporozoites, priming of CD8^+^ T cells may occur at two different anatomical locations, skin draining lymph nodes (DLNs) and the liver [5-7]. It was long assumed that activation of sporozoite-specific CD8^+^ T cells occurred in the liver. This idea was challenged when Zavala and colleagues, demonstrated that lymph nodes draining the infection site play a fundamental role in priming liver stage-specific CD8^+^ T cells. The authors observed a marked decrease in the number of activated circumsporozoite protein (CSP)-specific CD8^+^ T cells in the liver of mice treated with FTY720, which blocks T cell egress from lymph nodes [8], prior to injection of sporozoites, or following the removal of the skin DLN at the site of sporozoite inoculation [7]. These results demonstrated the importance of lymph nodes in mounting CD8^+^ T cell responses against *Plasmodium*. Furthermore, CD8^+^ T cell priming in skin DLNs is sufficient for the induction of protective immunity against sporozoite challenge [6].

A small fraction of sporozoites at the site of inoculation have been shown to mature into infectious merozoites [9], however it has also been shown that these skin exo- erythrocytic infections are not capable of initiating blood stage infections [10]. Alternatively, sporozoites can leave the infection site by entering either the blood or lymphatic circulation [11, 12]. Approximately 15–20% of the inoculum ends up in the skin DLN [6, 12]. Those sporozoites that reach the DLN represent a critical portion of the inoculum that primes CD8^+^ T cells. This is supported by the observation that 48 hours after inoculation of sporozoites, by either bites of irradiated infected mosquitoes or *via *intradermal inoculation, CSP-specific CD8^+^ T cells producing IFN-γ were only detected in the skin DLN [7]. Although analysis of sporozoite-specific CD4^+^ T cells, which can contribute towards control of liver stage parasites [13, 14], has not been determined, it is likely they are also induced in the skin DLN.

Once sporozoites enter lymph nodes, dendritic cells (DCs) phagocytose the parasites, and then process and present parasite antigens *via *cross-presentation [5, 7, 15, 16]. CD11c^+^DCs play a key role in the activation of *Plasmodium*-specific CD8^+^ T cells [7, 16], as *in vivo* depletion of these cells abolished the induction of parasite-specific CD8^+^ T cells [16]. However, the specific DC population responsible for the induction of parasite-specific CD8^+^ T cells is not known. There are multiple subsets of DCs in the dermis (e.g. resident dermal CD103^+ ^and CD11b^+^ DC subsets, Langer-hans cells or inflammatory monocyte-derived DCs) that are capable of cross-presenting viral antigens [17], and thus may be relevant in the activation of *Plasmodium*-specific CD8^+^ T cells. It is also possible that priming of parasite-specific CD8^+^ T cells requires a collaborative effort between skin migratory DCs and lymphoid-resident DCs [12, 18]. Thus, activation of *Plasmodium*-specific CD8^+^ T cells in skin DLNs might not rely on a single DC subset, but on the interaction of several DC populations.

As mentioned above, priming of *Plasmodium*-specific CD8^+^ T cells may also occur in the liver. Liver resident CD8α^+^CD11c^+^ DCs activate CD8^+^ T cells after immunization with irradiated sporozoites [5], and both liver sinusoidal endothelial cells and Kupffer cells are capable of processing and presenting antigens to naïve CD8^+^ T cells [19]. However, CD8^+^ T cells primed by liver antigen-presenting cells exhibit lower levels of activation (i.e., diminished expression of the activation markers CD44 and CD25) [19]. Finally, prolonged antigen presentation, following immunization with irradiated sporozoites, is also important in the optimal induction of sporozoite-specific CD8^+^ T cell responses [20]. Collectively, efficient generation of *Plasmodium*-specific effector CD8^+^ T cells, in rodent malaria, seems to be shaped by at least 3 factors (i) number of sporozoites inoculated into the host [21, 22], (ii) priming of CD8^+^ T cells in skin DLNs [7] and (iii) prolonged antigen presentation [20]. However, there are still many unknowns, including why natural infections and vaccines tested to date fail to induce protective liver stage-specific CD8^+^ T cell responses.

## REGULATION OF *PLASMODIUM*-INDUCED CD8^+^ T CELL ACTIVATION

The precise mechanism by which naïve *Plasmodium*-specific CD8^+^ T cells become activated is not clear, but clues are emerging. For example, it has been shown that NK cells, probably *via *IL-12, are necessary for optimal priming as depletion of NK cells significantly reduced CD8^+ ^T cell priming [23]. CD4^+ ^T cells also participate in generating liver stage-specific CD8^+^ T cells. Zavala and colleagues showed that in the absence of CD4^+^ T cells, CD8^+^ T cell responses are impaired as a consequence of undergoing premature contraction [24, 25]. Following sporozoite infection CD4^+^ T cells were shown to secrete IL-4, a cytokine with strong *in vivo* and *in vitro* anti-apoptotic effects on activated and resting CD8^+^ T cells [26], which signals directly to parasite-specific CD8^+^ T cells to help maintain a memory CD8^+^ T cell population [24]. Of note, these studies were conducted in BALB/c mice, which favor production of IL-4 and consequently Th2 biased responses. Thus, it will be important to determine whether the contribution of IL-4 to expansion of sporozoite-specific CD8^+^ T cells is universal (e.g., is it also important in C57BL/6 mice which favor production of IFN-γ and Th1 biased responses?) or a consequence of using BALB/c mice. In contrast to these signals that favor robust liver stage-specific CD8^+^ T cells, there are also negative signals that function to dampen CD8^+^ T cell responses. For example, activated CD8^+^ T cells can negatively regulate the subsequent activation of additional naïve CD8^+^ T cells *via *competition for antigen on antigen-presenting cells [27, 28]. Furthermore, skin CD4^+^ regulatory T cells (T_regs_) have been suggested to decrease expression of MHC class II and CD86 on skin DCs, which may impair activation of liver stage-specific CD8^+^ T cells [29]. Much of what we know about the precise mechanisms involved in the induction of liver stage-specific CD8^+^ T cells has been explored in the context of irradiated sporozoites. Thus, it is imperative we gain a better understanding of how liver stage-specific CD8^+^ T cells are generated following infection with live sporozoites *via *their natural route of infection and why *Plasmodium*-infected humans fail to induce protective CD8^+^ T cell responses in spite of repeated infections.

## MECHANISMS OF CD8^+ ^T CELL MEDIATED PROTECTION DURING THE LIVER STAGE

Once sporozoites enter the liver they glide along sinusoids where they ultimately invade and infect the liver parenchyma through an elegant process that has been reviewed elsewhere [30]. The liver stage of the *Plasmodium *life cycle is marked by an exponential expansion of parasite numbers and differentiation into merozoites that infect red blood cells when released from hepatocytes. It is estimated that one sporozoite can give rise to about 40,000 merozoites [31]. The liver stage of the life cycle is also relatively short, lasting about 2 days in mice [1], and about one-week in humans [2]. Thus, liver stage-specific CD8^+^ T cells must overcome substantial hurdles (i.e., the relatively few infected hepatocytes, the short duration of the liver stage, and the necessity to eliminate every infected hepatocyte) if they are to prevent the parasite from progressing from the asymptomatic liver stage to the symptomatic blood stage. Given the short duration of the liver stage, *Plasmodium*-specific CD8^+^ T cells primed during the initial exposure, which require one to two weeks for optimal expansion [32], likely contribute very little to liver stage immunity. However, liver stage-specific memory CD8^+^ T cells can play a critical role at controlling *Plasmodium* infected hepatocytes during a secondary infection [3, 33, 34].

Circulating memory CD8^+^ T cells can be broadly defined as either effector memory T cells (CD62L^lo^/CD27^lo^/IL-2^lo^) or central memory T cells (CD62L^hi^/CD27^hi^/IL-2^hi^) [33]. Consistent with enhanced protection mediated by effector memory T cells following infection with *Listeria monocytogenes* and lymphocytic choriomeningitis virus [35, 36], effector memory T cells also provide increased protection against *Plasmodium* infected hepatocytes compared to central memory T cells [37-39]. Nevertheless, central memory CD8^+ ^T cells correlate with sustained protection against malaria in mice [34], which is likely explained by the long-term stability of central memory CD8^+^ T cell numbers [40].

CD8^+^ T cells are endowed with multiple effector pathways, which include direct and indirect mechanisms, to eliminate target cells. In the case of liver stage-specific CD8^+^ T cells both are involved in controlling the parasite infection. Direct effector pathways used by* Plasmodium* liver stage-specific CD8^+^ T cells include the release of perforin and granzymes [27, 41], whereas indirect effector mechanisms include the production of IFN-γ and TNF [5, 42-44].

Among CD8^+ ^T cell effector mechanisms, IFN-γ is important for controlling infected hepatocytes [5, 42-44]. The exact mechanism by which IFN-γ exerts its protective effect against *Plasmodium* is not fully known, but probably involves multiple mechanisms. IFN-γ causes increased expression of MHC class I, which enhances the recognition of antigens by memory CD8^+ ^T cells [45]. Similarly, IFN-γ facilitates the conversion of the proteasome to the immune proteasome, which increases production of peptides that occupy MHC class I molecules [46, 47]. Another mechanism by which IFN-γ suppresses parasite development is through direct impairment of parasite differentiation in hepatocytes [48]. IFN-γ, from *Plasmodium*-specific CD8^+^ T cells, has also been suggested to increase expression of inducible nitric oxide synthetase, which results in increased production of nitric oxide that confers protection against *Plasmodium *[49], however the mechanism by which nitric oxide inhibits the development of liver stage parasites is not known.

Although production of IFN-γ may be the most critical mechanism by which CD8^+^ T cells eliminate infected hepatocytes, TNF also participates in *Plasmodium *control during the liver stage. For instance, *in vitro *administration of TNF prevents the development of human and rodent malaria pre-erythrocytic stages, but the mechanism of action of TNF against the parasite is unclear [4, 50]. However, an earlier study suggested TNF inhibits *P. yoelii *liver stages *in vitro* *via *synthesis of IL-6 [51], which shows anti-parasite activity potentially mediated by oxidative burst [52]. It was also shown that *in vivo* neutralization of TNF, *via *treatment with anti-TNF monoclonal antibodies, substantially reduced protection against either *P. berghei* or *P. yoelii* sporozoite challenge in a CD8^+^ T cell-dependent model [53].

There is also evidence to support a role for CD8^+^ T cells mediating pre-erythrocytic protection *via *direct cell contact. Following vaccination of mice with *P. yoelii* genetically attenuated parasites (GAPs) protection against subsequent *P. yoelii* sporozoite challenge is perforin-dependent [54]. The absence of perforin in memory CD8^+^ T cells also results in a 50% decrease in protection against *P. yoelii* sporozoite challenge in a prime-boost model that generates only *P. yoelii* CSP-specific CD8^+^ T cells [53]. However, the requirement for perforin in CD8^+^ T cell mediated protection in this model is species specific, as perforin-deficiency had no effect on protection against *P. berghei* sporozoite challenge [53]. Species-specific requirements for CD8^+^ T cell effector molecules was also noted in mice vaccinated with radiation-attenuated sporozoites (RAS) [13]. These data are also consistent with the observation that the numerical threshold of CSP-specific CD8^+^ T cells required for protection against sporozoite challenge is dependent on both *Plasmodium* species and the genetic background of the host [13, 39].

It is well established that* Plasmodium*-specific CD8^+^ T cells are able to prevent progression of *Plasmodium* infections from the pre-erythrocytic stage to the erythrocytic stage, however how such events occur needs to be elucidated. Cockburn and colleagues, provide useful clues using key developments in several technologies that allowed them to visualize *Plasmodium*-specific CD8^+^ T cells interacting with *Plasmodium* infected hepatocytes in mice in real time *in vivo*. Upon recognition of infected cells,* Plasmodium*-specific CD8^+^ T cells form large clusters around infected hepatocytes [55]. The formation of these cellular clusters may facilitate CD8^+^ T cells to eliminate parasites from the liver. The development of these technologies may contribute important findings as to how CD8^+^ T cells identify and eliminate infected hepatocytes.

## APPROACHES TO GENERATE PROTECTIVE CD8^+^ T CELLS

Given the protective capacity of *Plasmodium* liver stage-specific CD8^+^ T cells in rodents substantial research has been directed towards developing CD8^+^ T cell based vaccines. Currently, there are four approaches to generate *Plasmodium*-specific CD8^+^ T cells, which can provide complete or partial protection against *Plasmodium* sporozoite challenge. These techniques include the use of RAS, GAP, wild-type sporozoites with chemoprophylaxis (CPS) and viral vectors (VV) that express *Plasmodium *antigens. Attenuation of *Plasmodium* by either radiation or targeted gene deletion results in viable sporozoites that infect hepatocytes and subsequently arrest within the liver without progressing into the blood stage. Consequently, the host is exposed to the full complement of sporozoite antigens. Likewise, vaccination with wild-type sporozoites with CPS not only exposes the host to all sporozoite antigens, but also to antigens expressed during the liver and blood stage. This allows the host to mount a diverse immune response including CD8^+^ T cells, CD4^+^ T cells, and antibodies. In contrast, VV induce an immune response to a small subset of parasite antigens. Of note, a diverse immune response including CD8^+^ and CD4^+^ T cells and antibodies directed against both pre-erythrocytic and erythrocytic antigens will likely be necessary in the development of an efficacious vaccine against *Plasmodium*.

### Radiation-Attenuated Sporozoites

RAS have been used to induce sterilizing liver stage specific immunity not only in rodents [56] but also in humans [57]. RAS involve the application of radiation (gamma or X ray) to sporozoites, which leads to random DNA damage and impairs subsequent gene transcription [58]. Radiation-induced DNA damage does not alter the capacity of the parasite to infect hepatocytes, however the life cycle is arrested at early stages [59-61]. The level of protection by RAS is not influenced by the source of radiation, but the dose of radiation is critical [3, 59, 62]. Large doses of radiation may kill sporozoites and limit their ability to infect hepatocytes and induce protective immunity, while low radiation doses allow the parasite to complete the liver stage and progress into the blood stage. RAS induced protection is CD8^+ ^T cell-dependent [13, 53] and correlates with effector memory CD8^+ ^T cells [33]. Of note, use of RAS also induces sporozoite-specific antibodies that potentially contribute to protection, as well as CD4^+^ T cells that have been shown in some cases to provide protective immunity in mice [13, 63-65].

In spite of the technical hurdles associated with large-scale implementation of RAS vaccination, Hoffman and colleagues have made significant strides to make the RAS approach a practical vaccine procedure [66]. So far in fact, only the RAS approach [57] and a vaccine that uses immunogenic fragments of *Plasmodium falciparum* CSP known as the RTS,S vaccine [67, 68] have shown promise as human vaccines. RTS,S has reached large-scale phase III testing, however results have been disappointing and suggest the vaccine affords only limited protection (~16-40%) against severe disease while no protection against infection or mortality [69-71]. One hopes the RAS vaccination approach will prove to be more efficacious than RTS,S when fully tested.

### Genetically Attenuated Parasites

Over the last decade advances in *Plasmodium *genetics have resulted in the generation of parasites lacking genes necessary for completion of the liver stage [61]. Subsequent infection with sporozoites from these GAPs are as immunogenic as RAS, with protective immunity dependent on CD8^+^ T cells [54]. However, like RAS vaccination it is possible CD4^+^ T cells and antibodies also contribute to GAP induced protective immunity.

The specific gene or combination of genes deleted determines the point at which the parasite arrests during liver stage development [72]. Currently, ten genes (P36p/P36, UIS3, UIS4, E1α, E3, SAP1/SLARP, FABI, FABB/F, FABZ, and PKG) have been deleted to manipulate the life cycle of the parasite [61]. Deletion of P36p results in normal sporozoite motility and hepatocyte invasion, but causes early arrest of the parasite in the liver due to impaired formation of the parasitophorus vacuole (PV) [73, 74]. When sporozoite and liver-stage asparagine-rich protein (SLARP) in *P. berghei*, or its *P. yoelii *ortholog sporozoite asparagine-rich protein 1 (SAP1) are deleted, sporozoites can still invade hepatocytes and form the PV, but they do not progress further in the liver stage [75, 76]. Deletion of UIS3 and UIS4 arrests the differentiation from trophozoite into schizonts [77, 78]. In contrast to P36p and SAP1, deletion of genes associated with the fatty acid metabolism (E1α, E3, FABI, FABB/F, FABZ, and PKG) exhibit normal development until the final differentiation and release of merozoites [79-81].

Vaccination with GAPs that arrest early in the liver stage can induce protective immunity, but GAPs that arrest later during the liver stage are more effective [82]. One explanation for this difference is the increased antigen repertoire the host is exposed to in late arresting GAPs compared to early arresting GAPs [61]. This was shown by Butler and colleagues, who demonstrated superior protective immunity in mice vaccinated with late arresting GAPs, compared to either early arresting GAPs or RAS, was the result of a larger and broader CD8^+^ T cell response in late arresting GAP vaccinated mice compared to early arresting GAP or RAS vaccinated mice [83]. This observation is consistent with prior reports that demonstrate the magnitude of *Plasmodium* specific CD8^+^ T cells is important in providing protection against sporozoite challenge [39, 84]. Furthermore, late arresting GAPs generate a host immune response that exhibits cross-stage specificity targeting both the liver and blood stages [83].

### Wild Type Sporozoites with Chemoprophylaxis

The use of wild type sporozoites with CPS to stimulate the immune system is based on the administration of viable sporozoites in conjunction with anti-parasitic drugs to induce host immune responses while controlling the perpetuation of the parasite. So far several drugs have been used, which target the parasite at either the late liver or blood stages. Pyrimethamine, centanamycin and primaquine prevent nuclear division of liver schizont stages [85]. Azithromycin and clindamycin exert delayed action by directly inhibiting apicoplast maturation of liver schizont stages while chloroquine affects the blood stage of the parasite life cycle [86-88]. Consequently, chloroquine provides the latest arrest of the parasite life cycle of all the strategies mentioned above [86, 87], which increases the number of parasite antigens the host is exposed to. Furthermore, chloroquine mediated CPS and late arresting GAP immunization strategies may also afford enhanced protection as a consequence of increased parasite biomass.

Vaccination with viable sporozoites under CPS is very similar to either RAS or GAP vaccination. However, protective immunity in humans was induced following exposure to just 10 bites from infected mosquitoes [89] while protective immunity elicited by RAS in humans required the bites of >1000 infected mosquitoes [90, 91]. Whether this differential outcome is associated with exposure to blood stage antigens following sporozoite infection and CPS compared to RAS is not known, but these results clearly highlight the potency of this approach over RAS. Although vaccination of viable sporozoites under CPS induces CD4^+^ T cell and antibody responses, protection in humans correlates with liver stage-specific CD8^+^ T cells [42].

### Subunit Vaccines

Viral vectors have been extensively evaluated as malaria vaccine candidates based on their ability to encode *Plasmodium* antigens and induce subsequent CD8^+^ T cell responses [92]. Examples of viral vector platforms for inducing *Plasmodium*-specific CD8^+^ T cell responses include replication-deficient adenoviruses (e.g., human, simian, and chimpanzee serotypes) and replication-deficient orthopoxviruses (e.g., modified vaccinia virus Ankara (MVA) and fowl pox 9 virus) [93, 94]. In addition, alphavirus, flavivirus, and morbillivirus may represent platforms to generate *Plasmodium*-specific CD8^+^ T cell responses [94]. In comparison with the other techniques used for induction of *Plasmodium*-specific CD8^+^ T cell responses, viral vectors overcome many manufacturing complications related with mosquito and/or sporozoite based formulations [95]. Another advantage of viral vectors is their ability to carry multiple transgenes and immune-stimulatory molecules, such as TLR agonists [96]. They also afford the ability to introduce cross-stage antigens to induce both liver and blood stage immune responses.

One of the main limitations of viral vectors is pre-existing immunity to the viral vector itself, which can dampen the host immune response to the transgene [97, 98]. Furthermore, as a consequence of the host immune system responding to the viral vector, it is essential that subsequent booster immunizations be done with different viral vectors engineered to carry the same *Plasmodium *transgene. Of the viral vectors evaluated as candidate malaria vaccines, poxviruses have been the most extensively studied in the clinic, however they have provided only modest protection against sporozoite challenge in humans [99, 100]. Consequently, chimpanzee adenoviruses have been targeted based on their ability to both prime robust CD8^+^ T cell responses and avoid pre-existing vector immunity [101].

## ROLE OF CD8^+ ^T CELLS DURING THE BLOOD STAGE OF *PLASMODIUM*

Experimental models have demonstrated CD8^+^ T cells are important in the immune response against liver stage parasites. In contrast, they contribute little to protective immunity during the blood stage [102, 103] and are potentially pathogenic [104-106]. The limited role of CD8^+^ T cells during the blood stage *Plasmodium* infection is explained by the lack of MHC class I on the surface of infected red blood cells [107]. Although blood stage-specific CD8^+^ T cells contribute little to protective immunity they are efficiently primed during infection in a process that involves cross-presentation mediated by CD8α^+ ^DCs [108]. In contrast, it has been reported that IL-10 impairs the ability of DCs to fully prime CD8^+^ T cells during malaria, resulting in decreased proliferation and cytokine production [109]. Regardless of their induction, there is strong support for blood stage-specific CD8^+^ T cells contributing to *Plasmodium*-induced pathology during experimental cerebral malaria in mice [104, 110-113]. There appears to be at least two mechanisms by which this occurs. First, CD8^+^ T cells, through an unknown mechanism involving IFN-γ, contribute to parasite accumulation in the brain [114]. Second, following recognition of antigen, *Plasmodium*-specific CD8^+^ T cells release perforin and granzyme B, which leads to experimental cerebral malaria [112, 115]. Curiously, CD8^+^ T cells do not recognize parasite infected RBCs, thus it’s not clear what cells stimulate the CD8^+^ T cells to release perforin and granzyme B. One possibility is that vascular endothelial cells acquire antigen from infected RBCs during cytoadherance, which is then recognized by CD8^+^ T cells. Of note, it is unknown if CD8^+^ T cells contribute to cerebral malaria in humans.

## CD8^+^ T CELL MEDIATED IMMUNITY AGAINST *TOXOPLASMA GONDII*

*Toxoplasma gondii *is the causative agent of toxoplasmosis, its an apicomplexan parasite that infects a wide range of vertebrates including humans [116, 117]. This parasite is transmitted between animals by ingestion of oocysts found in feline feces or tissue cysts in infected vertebrates [118]. Once in the intermediate host, the parasite undergoes asexual replication and disseminates throughout the body, including the brain, where it establishes intracellular infections and the formation of cysts [118]. Control of *T. gondii *requires the synergic interaction of multiple soluble (i.e. IL-12, IFN-γ) and cellular components (natural killer cells, DCs, macrophages, and CD4^+^ and CD8^+^ T cells) of the host immune system [119].

Depletion of CD8^+^ T cells, but not CD4^+^ T cells, using monoclonal antibodies accelerates the mortality of mice chronically infected with *T. gondii *[120-122], which provides support for the role of CD8^+^ T cells in controlling the parasite during the chronic phase of the infection. CD8^+^ T cell mediated control of *T. gondii *is dependent upon the production of IFN-γ [123, 124] and perforin [125-127]. One mechanism by which IFN-γ contributes to the control of *T. gondii *is by stimulating monocytes, macrophages and non-hematopoietic cells to produce nitric oxide [128, 129], however the precise mechanism by which these molecules afford protection is not known. Both IL-2 and CXCL10 also contribute to CD8^+^ T cell control of *T. gondii*. IL-2, which is produced by CD8^+^ T cells, increases IFN-γ production during the secondary response to *T. gondii *through an autocrine feedback loop [124], while CXCL10 was demonstrated to maintain effector CD8^+^ T cells in the brain and regulate migration speed towards *T. gondii* infected cells [130].

## ROLE OF CD8^+^ T CELLS DURING *LEISHMANIA* INFECTION

Leishmaniasis is caused by various species of *Leishmania *(*L. major*, *L. donovani*, *L. braziliensis, L. infantum, etc.*), and is transmitted by 30 different sand fly species [131]. Clinical manifestations range from self-healing cutaneous lesions to deadly visceral disease. The contribution of CD8^+^ T cells in mediating protection against experimental cutaneous Leishmanias has been controversial. Early evidence suggested CD8^+^ T cells contributed to protective immunity [132]. However, subsequent studies identified CD4^+^ Th1 cells as the primary cells involved in controlling infection [133]. These contradictions were later resolved when it was shown that following low dose infection CD8^+^ T cell produced IFN-γ was necessary for the development of Th1-polarized CD4^+^ T cells, while after high dose infection CD8^+^ T cells were not required for the generation of a protective Th1 response [134]. In addition to their role in cutaneous Leishmaniasis, CD8^+^ T cells also provide protection against visceral Leishmaniasis. During visceral Leishmaniasis CD8^+^ T cells aid in the development of granulomas in the liver of infected mice [135], and the reduction of parasite burden in the spleen [136]. Moreover, CD8^+^ T cells have been shown to contribute to protective immunity during secondary *Leishmania* infections in mice [137]. The insights learned from experimental Leishmaniasis appear to hold up in humans, where CD8^+^ T cells also correlate with protective immunity (reviewed by Stagar and Rafati [138]).

## ROLE OF CD8^+^ T CELLS DURING *TRYPANOSOMA CRUZI* INFECTION

*T. cruzi* is the causative parasite of Chagas disease, a zoonotic chronic inflammatory disease transmitted by haematophagous triatomine insects [139]. CD8^+^ T cells contribute to control of the parasite during acute and chronic stages of the disease [140-142]. A role for CD8^+^ T cells in the control of acute *T. cruzi *infection was shown through the use of β2-m-deficient mice, which lack MHC class I expression on the cell surface, as these mice succumb to acute infection [143]. Additionally, CD8^+^ T cells are required for control of chronic *T. cruzi* infection as depletion of CD8^+^ T cells during chronic infection resulted in exacerbation of inflammation within the heart, the site of chronic infection, and an increase in parasite burden [144]. Moreover, recent work has identified a number of CD8^+ ^T cell epitopes within the *T. cruzi* genome, including an immune dominant epitope located in the trans-sialidase gene [145-148]. Identification of these epitopes may facilitate additional studies to evaluate the contribution of CD8^+^ T cells to protective immunity against *T. cruzi* and may also guide sub-unit based vaccines against this parasitic infection.

In spite of the induction of *T. cruzi*-specific CD8^+^ T cells there are several notable abnormalities associated with this response, which may contribute to impaired clearance of the parasite and progression to a chronic infection. For example, expansion of CD8^+^ T cells is delayed and remains relatively low in numbers during the first week of infection [148-150], which likely contributes to dissemination and an increase in the parasite burden throughout the host. Effector and memory CD8^+^ T cells accumulate at the site of infection [151], however effector functions of CD8^+^ T cells are attenuated and the CD8^+^ T cells eventually become exhausted [151-154]. Given the impact of *T. cruzi* on human health there is still much to be learned about the host immune response, including CD8^+^ T cells, to this parasite.

## CONCLUSION

CD8^+^ T cells contribute to protective immunity against multiple intracellular parasitic infections. In recent years we have learned a great deal about how CD8^+^ T cells are primed, expand into effector and memory populations, and contribute to protective immunity against *Plasmodium* spps. and *T. gondii*. However, the contribution of CD8^+^ T cells in host immune responses to *Leishmania* and *T. cruzi* are not as well defined. A greater understanding of the requirements for CD8^+^ T cells to mediate protective immunity against these parasitic infections, especially in humans, is needed in order to develop effective vaccines against these pathogens.
